# Essential role of a *Plasmodium berghei* heat shock protein (PBANKA_0938300) in gametocyte development

**DOI:** 10.1038/s41598-021-03059-4

**Published:** 2021-12-08

**Authors:** Mohammad Kashif, Afshana Quadiri, Agam Prasad Singh

**Affiliations:** Infectious Diseases Laboratory, National Institute of Immunology, Aruna Asaf Ali Marg, New Delhi, 110067 India

**Keywords:** Biotechnology, Cell biology, Genetics, Microbiology

## Abstract

The continued existence of *Plasmodium* parasites in physiologically distinct environments during their transmission in mosquitoes and vertebrate hosts requires effector proteins encoded by parasite genes to provide adaptability. Parasites utilize their robust stress response system involving heat shock proteins for their survival. Molecular chaperones are involved in maintaining protein homeostasis within a cell during stress, protein biogenesis and the formation of protein complexes. Due to their critical role in parasite virulence, they are considered targets for therapeutic interventions. Our results identified a putative *P. berghei* heat shock protein (HSP) belonging to the HSP40 family (HspJ62), which is abundantly induced upon heat stress and expressed during all parasite stages. To determine the role HspJ62, a gene-disrupted *P. berghei* transgenic line was developed (ΔHspJ62), which resulted in disruption of gametocyte formation. Such parasites were unable to form subsequent sexual stages because of disrupted gametogenesis, indicating the essential role of HspJ62 in gametocyte formation. Transcriptomic analysis of the transgenic line showed downregulation of a number of genes, most of which were specific to male or female gametocytes. The transcription factor ApiAP2 was also downregulated in ΔHspJ62 parasites. Our findings suggest that the downregulation of ApiAP2 likely disrupts the transcriptional regulation of sexual stage genes, leading to impaired gametogenesis. This finding also highlights the critical role that HspJ62 indirectly plays in the development of *P. berghei* sexual stages and in facilitating the conversion from the asexual blood stage to the sexual stage. This study characterizes the HspJ62 protein as a fertility factor because parasites lacking it are unable to transmit to mosquitoes. This study adds an important contribution to ongoing research aimed at understanding gametocyte differentiation and formation in parasites. The molecule adds to the list of potential drug targets that can be targeted to inhibit parasite sexual development and consequently parasite transmission.

## Introduction

*Plasmodium* belongs to phylum protozoa and causes one of the most devastating diseases, is responsible for millions of clinical cases, and affects approximately 1.2 billion lives worldwide^1^. The *Plasmodium* life cycle is completed in two physiologically distinct habitats that include cold-blooded invertebrate vectors and warm-blooded vertebrate hosts. Even within the vertebrate host, the parasites encounter two different cellular environments, i.e. hepatocytes and erythrocytes^2,3^. The continuously changing physiological environment creates stressful conditions for the parasite^4^. Parasites require an intrinsic adaptation to manage the robust changes they confront while cycling between mosquitoes and their hosts.

Elevated temperature of the infected host may lead to numerous changes within the cell, including protein denaturation and aggregation, changes in membrane fluidity and, in extreme cases, may even cause cell cycle arrest^5,6^. The malaria parasite also encounters considerable oxidative stress during its asexual erythrocytic stage due to heme detoxification, cell metabolic processes, and the immune responses induced in the host^7^. In addition, the *Plasmodium* proteome has aspargine (Asn) repeat-rich sequences, which increases the tendency of proteins to aggregate^8^. Protein aggregation and unfolding are further amplified during heat stress. Therefore, the parasite induces the expression of HSPs in response to cellular stress. These proteins are expressed upon stress as well as during normal developmental processes^6,9^.

The crucial mediators of the heat shock response during elevated temperatures in the host are heat shock proteins (HSPs) that display stress-induced expression. As molecular chaperones, these proteins promote the folding of cellular proteins and prevent their aggregation^10^. Approximately 2% of the *Plasmodium* genome codes for molecular chaperones^11^. Chaperones are a family of proteins responsible for proper folding of translated peptide chains into their monomeric or oligomeric forms during stress^12^. Based on their molecular weight, HSPs are categorized into eight main families: HSP110, HSP100, HSP90, HSP70, HSP60, HSP40, HSP10, and small HSPs (sHSPs)^13^. Comparison with other organisms shows that more than 50% of HSP proteins (~ 43) in the *Plasmodium* parasite belong to the HSP40 family^14^. Based on their conserved domains, the HSP40 protein family is divided into four distinct classes: Type I HSP40 proteins contain an N-terminal J-domain, zinc-binding cysteine rich domain, substrate-binding C-terminal, histidine-proline-aspartate (HPD) motif, and glycine/phenylalanine (GP) rich region. The GP region separates the N-terminal J domain from the rest of the protein. Type II proteins do not have zinc-binding domains, while only DNAJ domains are present in type III and IV HSP40 proteins; however, type IV protein sequences exhibit disparities in the HPD motif^14–16^. Generally, HSP families are conserved in *Plasmodium* species, while comparative data analysis of the molecular chaperone families shows that there are some members that are specific to stage^17–19^, lineage^20^ or species^21^ of *Plasmodium*. RESA is a type IV Hsp40-like protein that is expressed in parasites during early development of the merozoite stage and interacts with the membrane of invaded erythrocytes to prevent further invasion^22^. Out of 43 Hsp40 proteins, 19 proteins show homology with their orthologues from other species of Apicomplexan, indicating that these proteins could have specific functions in the parasite and could be promising drug targets, while variance was observed in most of the exported proteins^14^.

Heat shock-induced responses are transitory and are removed as soon the stress is controlled. Hsp70 actively acts as a chaperone with the assistance of cochaperones or Hsp40 partners, which adequately stimulate its ATPase activity and promote its interaction with translated polypeptide substrates. Studies show that some HSP40 family proteins are heat-inducible and colocalize with the HSP70-1 family, and their expression increases with temperature and environmental stress^23^. Cochaperone HSP40 family proteins regulate the chaperone activity of the HSP70 and HSP90 protein families by assisting in the folding of proteins under both stress and normal conditions^15,16,24–26^. Studies have shown the role of HSPs in the modulation of DNA-binding activity within the nucleus and the survival of parasites against host defenses^27^. Certain HSPs are expressed selectively in the nuclei of merozoites and schizonts and are believed to be involved in signal transduction for transcription and the expression of genes encoding proteins essential in the defense of *Plasmodium* parasites against the host immune system and other responses to which the parasites are exposed in the host. The molecular cochaperone HSP40 family protein also refers to DnaJ proteins, assists in regulating protein folding, protein trafficking, assembling and disassembling of protein complexes, and translocates different proteins to distinct subcellular compartments^28,29^.

Molecular chaperones and their cochaperones play major roles in the folding, translocation, and degradation of proteins, indicating that they are essential for the maintenance of cellular proteostasis. The parasite’s ability to transport its proteins to the RBC surface, which aids in adhesion of invaded erythrocytes to the blood vessels, leads to malaria pathogenesis. Host chaperones exist in association with parasitic proteins at the erythrocyte membrane^30^. This implies that host chaperones assist the trafficking of parasite proteins to the erythrocyte membrane. The chaperones eliminate cellular stress by helping correctly fold proteins and inhibiting their misfolding or aggregation. Host factors, especially host immunity and temperature generated by periodic fever in malaria patients, influence the rate of gametocyte production. *Plasmodium* gametogenesis was observed to be induced as a stress response of the parasite to increasing temperature and deteriorating conditions in the infected blood^31^. The life cycle of *Plasmodium* parasites involves extensive physiological and morphological changes, which also requires the systematic control of protein expression. The regulation of heat shock proteins thus has a prominent role in *Plasmodium* development. The proteins of the Hsp40 family display greater structural variety than their Hsp70 counterparts^32^. Furthermore, the same Hsp70 can be regulated by different Hsp40 family members, leading to different responses of Hsp70. Considering the regulatory role of HSP40 family proteins, we investigated the role of *Plasmodium* HspJ62 in the parasite using two different approaches: gene knockout and protein–protein interaction methodologies.

Comparative analysis of human and *Plasmodium* genomes has revealed certain unique characteristics possessed by parasite proteins. Such differences can be exploited for developing drugs against malaria. The characterization of HSP proteins and their localization and interaction with cochaperones will enhance our understanding of parasite biology and help us to develop assays and find molecules for targeting the parasite. In this regard, we aimed to characterize a *Plasmodium berghei* HSP molecule (PBANKA_0938300) referred to as HspJ62 in our subsequent studies. HspJ62 protein is a putative heat shock protein containing a DNAJ domain. One of the reasons for choosing this molecule from the pool of HSP40 molecules was that it is a putative heat shock protein, is a novel molecule and was not functionally characterized. We observed by manually analyzing the protein sequence that the protein had two putative Plasmodium Export Element (PEXEL) motifs^33^ (Supplementary Fig. 3A, boxed amino acid sequence). Although the functional validation of these putative domains has yet to be confirmed, the presence of these domains in the sequence indicated that the protein is likely exported to the host cytoplasm. *Plasmodium* proteins containing PEXEL are a five-residue peptide sequence motif frequently found in parasite proteins that are transported beyond the confines of the vacuolar membrane. Our study sheds light on the characterization of the HspJ62 protein and its role in the development of the sexual stage of *Plasmodium* parasites. Here, we demonstrate that HspJ62 is essential for sexual stages of the parasite life cycle.

## Result

### HspJ62 is induced under heat stress condition

**Figure 1:**
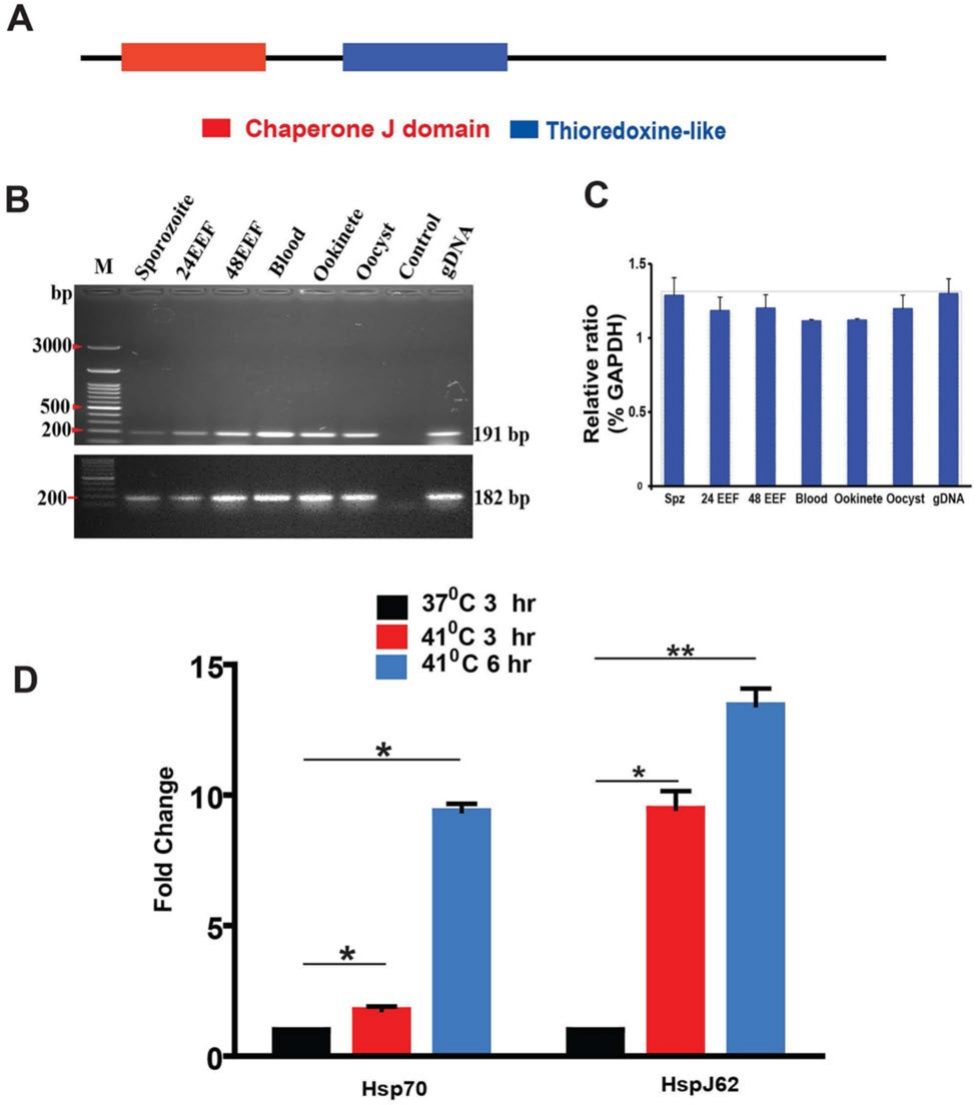
HspJ62 is induced under heat stress conditions. (**A**) In silico analysis of the functional domains of HspJ62 shows that the DnaJ domain (34–93 amino acids) and thioredoxine-like domain (188–289 amino acids) are typical of the Type II HSP40 protein family. (**B**) The result of PCR using cDNA synthesized from RNA isolated from different stages of parasite is shown. PCR analysis shows the presence of gene transcripts throughout the life cycle of parasites. Genomic DNA (gDNA) was used as a positive control, while water was used in place of a template in the negative control; EEF: exoerythrocytic forms 24 and 48 h post infection; Blood: mixed blood stages. Full blots are shown in Supplementary Fig. 5A and B. (**C**) Bands present in figure A were quantified by densitometry and normalized to *Gapdh* and plotted as graph. (**D**) Quantitative PCR using RNA extracted from blood stage parasites cultured at normal 37 °C and higher 41 °C temperatures. The transcript levels were normalized to the *Gapdh* housekeeping gene, and *Pb*Hsp70-1 transcript was included as a positive control. The data are shown as the gene expression at 41 °C compared to 37 °C and represent the mean value of two separate experiments performed in triplicate along with standard error of the mean (SEM) shown as error bars. Statistical analysis was performed using the unpaired T-test; **P < 0.01, *P < 0.1.

We initiated the characterization of HspJ62 by in silico analysis. The P-fam prediction tool showed that HspJ62 belongs to the Type II HSP40 protein family and has a DnaJ domain and thioredoxine-like domain (Fig. 1A). HSP proteins are induced during cell stress to promote correct folding and functioning of proteins. To understand the expression of HspJ62 during the *Plasmodium* life cycle, HspJ62 transcripts were analyzed during different parasitic stages. For this, total RNA was isolated from different parasitic stages, including sporozoite, exoerythrocytic stages at 24 and 48 h post HepG2 infection (HepG2 cells were infected with sporozoites, and cells were harvested at 24 and 48 h post infection), blood stages, and mosquito stages. End point PCR using HspJ62 gene-specific primers and GAPDH primers as loading controls was conducted. GAPDH primers give 182 bp PCR product. PCR showed that gene-specific transcripts (191 bp) were present in all stages of the parasite life cycle, demonstrating that the HspJ62 gene displays constitutive expression throughout the different stages of the *Plasmodium* life cycle (Fig. 1B and C, full blot given in Supplementary Fig. 5).

To investigate the expression of HspJ62 at elevated temperatures, *Plasmodium berghei*-infected blood was isolated from infected mice and grown in vitro at a physiological temperature of 37 °C and at a higher temperature of 41 °C separately. The parasites were harvested at 3 and 6 h from in vitro cultures. These time points were selected considering the duration of feverish sickness in patients, which is typically between 3 and 6 h during a primary infection. Quantitative PCR was conducted using the total RNA isolated from the harvested parasites. The results showed a considerable increase in the expression of HspJ62 at 41 °C. The transcription of HspJ62 was normalized using parasite 18S rRNA and *GAPDH* genes; Hsp70-1 (PBANKA_0711900) was included as a positive control (Fig. 1D and Supplementary Table 1). The gene exhibited expression (transcripts) corresponding to an increase of approximately 10- and 14-fold at 41 °C after 3 and 6 h, respectively. The resulting induction was significantly higher than the induction of housekeeping gene transcripts, indicating that it has an essential function as an HSP protein.

### HspJ62 protein purification and antibody generation

To explore the expression and localization of HspJ62 at the protein level, the recombinant HspJ62 protein was expressed with GST-tagged protein in *E. coli* and purified by affinity chromatography using GST resin. The recombinant HspJ62 protein has a molecular weight of approximately 72 kDa, as revealed by SDS-PAGE analysis. This was consistent with the expected molecular size of the HspJ62-GST fusion protein (Supplementary Fig. 1A). Due to the presence of the GST tag, the protein is immunoreactive to the anti-GST antibody. Therefore, recombinant HspJ62 expression was confirmed by immunoblotting using an anti-GST antibody (Supplementary Fig. 1D) and by mass spectrometry (Supplementary Fig. 2A). The purified recombinant protein was used to immunize rats to obtain anti-HspJ62 immune sera following the immunization regimen shown in Supplementary Fig. 1B. The titer of anti-HspJ62 antibody obtained from immunized rats was measured via ELISA. The antibodies could recognize the protein even at a dilution of 1:120,000, indicating the induction of a strong antibody response against recombinant HspJ62 (Supplementary Fig. 1C). The specificity of the antibody was established by employing immunoblotting using parasite lysates. The uninfected RBCs lysate was used as control. Antibodies recognized only the native protein in parasite lysates (Supplementary Fig. 1E).

**Figure 2:**
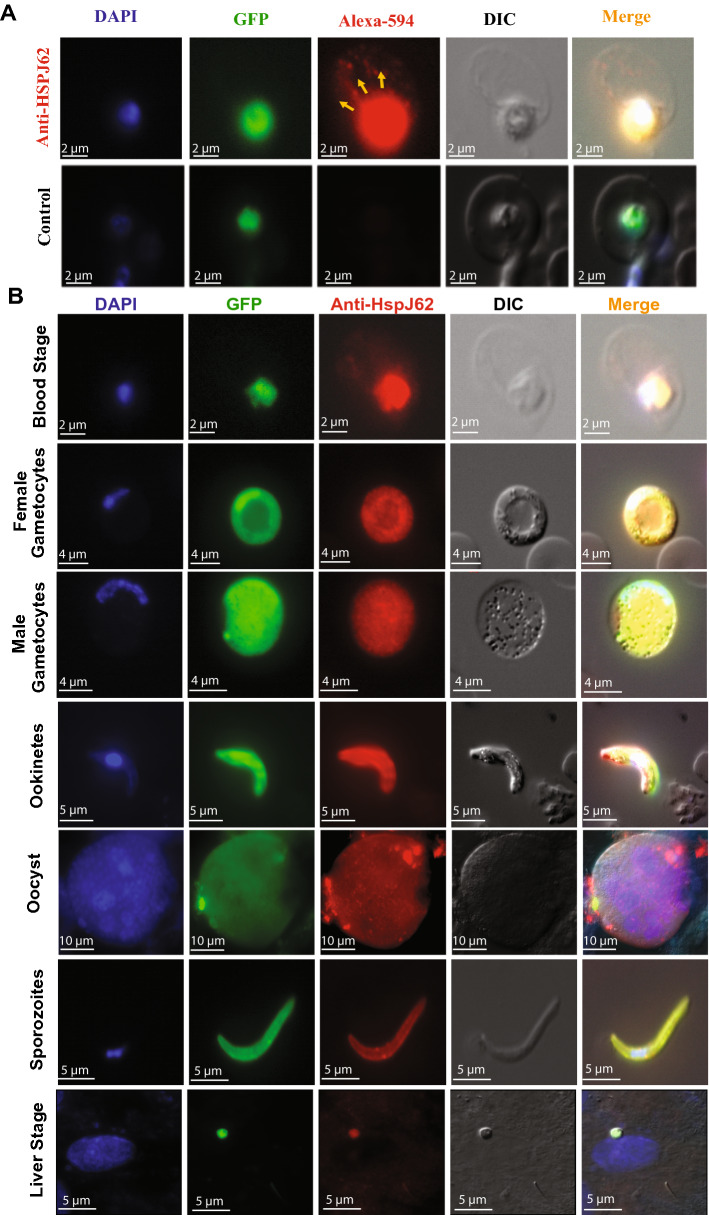
HspJ62 is expressed throughout the different stages of the parasite life cycle. (**A**) Representative cells show the export of protein across the PV membrane into the host cytosol. An anti-HspJ62 polyclonal antibody was used as the primary antibody, followed by an Alexa 594-conjugated secondary antibody. The parasite nuclei were stained with DAPI (blue), and the slides were visualized under a fluorescence microscope. Preimmune rat sera were used as a negative control. (**B**) Immunofluorescence assays to localize HspJ62 in the different developmental stages of parasites using anti-HspJ62 antibodies. The WT-GFPcon parasites (green) were used for IFA, and an anti-rat Alexa 594-conjugated secondary antibody (red) was used to detect the expression and localization of HspJ62 in all stages of *P. berghei*. The parasite nuclei were stained with DAPI (blue), and the slides were visualized under a fluorescence microscope. Preimmune rat sera were used as a negative control (Supplementary Fig. 2B).

The stage-specific expression of HspJ62 protein was checked by immunofluorescence assay using anti-HspJ62 antibody and a fluorescence microscope. The results demonstrated that the HspJ62 protein resides in parasites and is possibly exported into the RBC cytosol (Fig. 2A). Preimmune rat sera were used as a negative control. HspJ62 expression was detected in all parasitic stages (exoerythrocytic, erythrocytic and mosquito stages) using an anti-HspJ62 antibody (Fig. 2B and Supplementary Fig. 3B). However, the subcellular localization of HspJ62 in parasites has yet to be confirmed. Fluorescence was not detected in the parasitic stages when preimmune rat sera were used as the primary antibody (Supplementary Fig. 2B).

### Absence of HspJ62 protein impairs gametocyte formation

**Figure 3:**
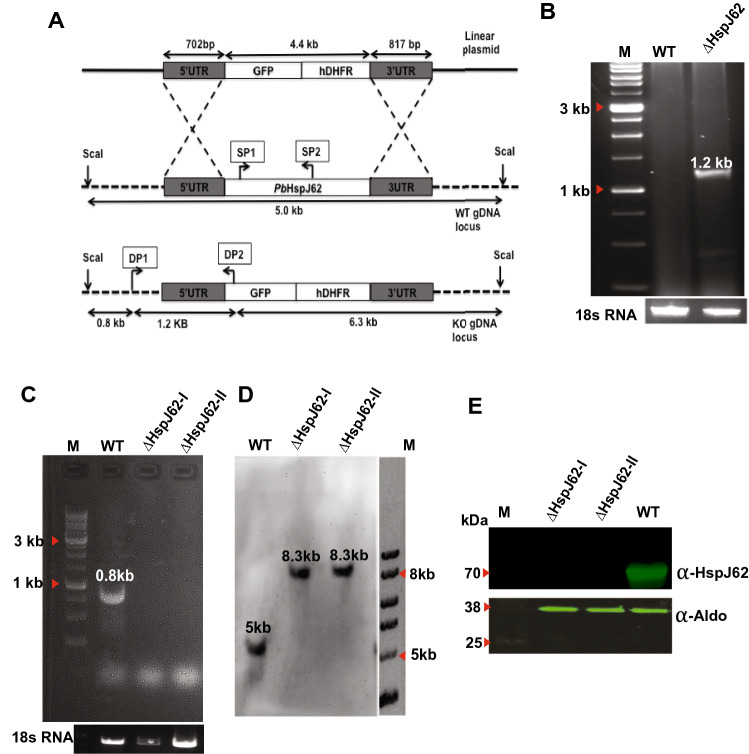
Generation and validation of the *P. berghei* HspJ62 knockout strain (ΔHspJ62). (**A**) The image shows the schematic summary of the HspJ62 gene knockout strategy by double homologous recombination using linearized vector-pBC-GFP-hDHFR-HSPJ62KO. The replacement cassette consisted of GFP and the hDHFR gene flanked by the 5’UTR (702 bp) and 3’UTR (817 bp) of the HspJ62 gene. (**B**) The expected product size of 1.2 Kb from diagnostic PCRs was observed. The external primer DP1 anneals to the chromosome, whereas DP2 is specific to the cassette. This primer pair shows specific band size 1.2 Kb only in the KO population. Full blots are shown in Supplementary Fig. 6A. (**C**) The two internal primers SP1 and SP2 anneal to the HspJ62 genomic locus. The PCR shows a specific band size of 842 bp only in the wild type and not in the knockout parasites. Full blots are shown in Supplementary Fig. 6B. (**D**) Southern blot analysis of ScaI-digested genomic DNA, obtained from WT and ΔHspJ62-I & II knockout parasite lines, using the 5’UTR of the HspJ62 gene as a probe resulted in the expected 5 Kb band corresponding to the WT gene and 8.3 Kb band corresponding to ΔHspJ62 parasite line. (**E**) Immunoblot analysis shows the absence of HspJ62 protein (62 kDa) expression in the ΔHspJ62-I & II parasite line compared with the WT parasite line. *Pb-*aldolase protein was used as a loading control. Full blots are shown in Supplementary Fig. 5F–H. *WT* wild type parasite lysate, ∆HspJ62-I and ∆HspJ62-II- are the parasite lysates from the two HspJ62 knockout parasite lines.

To determine whether HspJ62 is critical for *P*. *berghei* development, we generated an HspJ62 knockout (ΔHspJ62) parasite line. This was done by employing a homologous double crossover strategy wherein the HspJ62 gene was replaced by a targeting construct containing a pyrimethamine drug selection cassette and GFP reporter marker cloned between the flanking untranslated regions of the gene (Fig. 3A). Independent transfections were performed to delete the gene. Successful gene deletion by double crossover recombination was validated by diagnostic PCR using the primer pair DP1 and DP2 (Supplementary Table 1) specific to the knockout line. The primers gave the expected amplification corresponding to a band size of 1.2 kb specific to the ΔHspJ62 clonal population (Fig. 3B, full blot given in Supplementary Fig. 6). Gene deletion was also confirmed by using the gene-specific primers SP1 and SP2 (Supplementary Table 1), which gave a band size of 842 bp specific to the gene in the wild-type population only (Fig. 3C, full blot given in Supplementary Fig. 6). Gene deletion was further confirmed by Southern blotting using a 5’UTR probe that gave 5013 bp and 8350 bp bands in WT and ΔHspJ62, respectively, in ScaI-digested gDNA of WT and ΔHspJ62 parasite lines, revealing that the clonal knockout strains lacked the HspJ62 gene, as expected (Fig. 3D, full blot given in Supplementary Fig. 5). Gene deletion was further confirmed at the protein level by western blotting. The membrane was probed with rat anti-HspJ62 antibody followed by detection with IRDye 800CW (goat anti-RAT Ig-G, cat# 926-32219, LI-COR) and scanning with a LI-COR Odyssey classic imaging system. Parasite aldolase protein detected by anti-aldolase antibodies was used as a loading control (Fig. 3E). These results confirmed the gene deletion of HspJ62 in two clones, which were designated ΔHspJ62-I and ΔHspJ62-II and were used for further phenotypic characterization.

**Figure 4:**
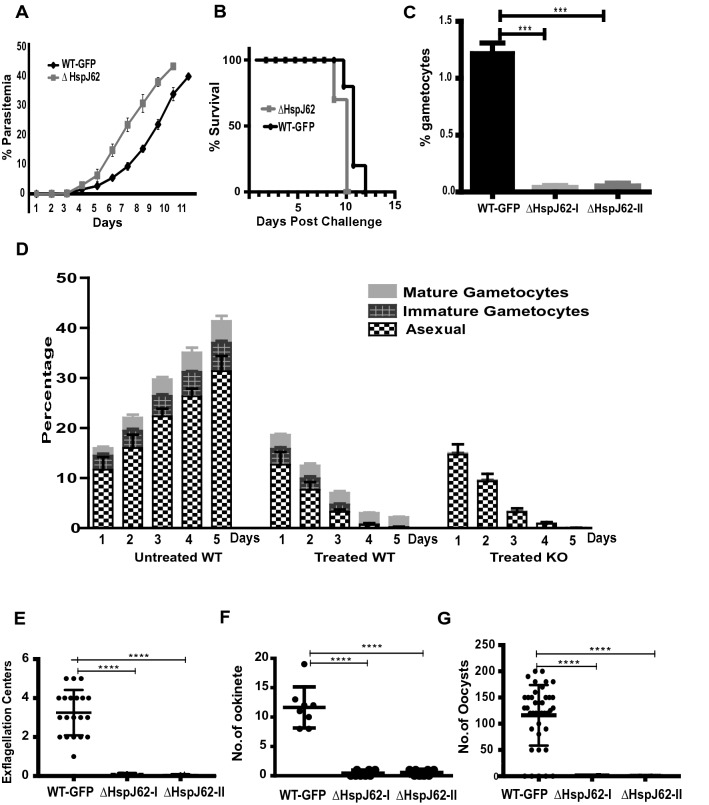
HspJ62 knockout parasites are unable to develop sexual stages. (**A**) The blood stage growth curves of the ΔHspJ62 parasite line in comparison with the wild-type strain are shown. Parasitemia was monitored daily from Day 1 until the mice died by Giemsa staining on slides, following intravenous injection of an equal number of WT or ΔHspJ62 parasites. (**B**) The survival of WT or ΔHspJ62 parasite-infected mice was counted and plotted. All data points are from one of the two representative experiments, showing the average daily parasitemia ± SD of groups of 10 mice each infected with WT or one of the two independent clones of ΔHspJ62 parasite (**A**, **B**). Gametocyte formation was monitored in wild-type and ΔHspJ62 parasites following intravenous injection of an equal number of WT or ΔHspJ62 parasites. (**C**) Gametocytemia was determined per 1000 red blood cells in Giemsa-stained blood smears. The graph shows the percent gametocytes formed in the clonal ΔHspJ62 parasite line in comparison with the wild-type strain; the error bars represent the mean and SD from three independent biological experiments conducted in triplicate. ***P < 0.0002, using one way ANOVA-Kruskal–Wallis test. (**D**) The effect of chloroquine (10 mg/ml) on WT and two independent clones of ΔHspJ62 parasite lines. (**E**) Exflagellation center formation was examined 5 min after activation of iRBCs collected from mice infected with WT-GFP or ΔHspJ62 parasites at day 4 post infection. The number of exflagellation centers was calculated from the number of actively moving gametes interacting with neighboring RBCs in 1000 RBCs. (**F**) The zygote/ookinete conversion rate is the percentage of female sexual stage parasites developing into zygotes or ookinetes. The zygote/ookinete conversion rate is generally calculated as the number of zygotes/ookinetes divided by the total number of female gametocytes. Since no male/female gametocytes were observed in ΔHspJ62 parasites, we looked for the formation of ookinetes per 10,000 RBCs. The number of ookinetes formed in WT and two independent clones of ΔHspJ62 parasites was calculated and plotted. (**G**) Oocyst formation and numbers were observed and counted in the mosquito midgut 14 days after an infected blood meal. Statistical analysis was performed using one-way ANOVA ****P = 0.0001. The image is representative of one of the three replicates performed.

For the phenotypic characterization of knockout parasites, approximately 1 × 10^4^ asexual stage parasites (ΔHspJ62-I and ΔHspJ62-II parasite lines) were injected via intravenous (i.v.) route in C57BL6 mice, and parasitemia was followed every day from the next day until the mice died. Giemsa-stained slides were observed under a bright field microscope. The knockout strains showed normal growth and morphology during asexual erythrocytic-stage development, similar to the wild-type line. Nevertheless, we observed higher growth rates for knockout clones than for the wild type (Fig. 4A). Since the knockout parasites multiplied faster, infected mice also showed reduced survival of 2 days in comparison to wild-type mice (Fig. 4B). To understand the reason for the high parasitemia in ΔHspJ62 parasites, we investigated gametogenesis. In the blood smears, we observed that sexual stages were affected in knockout, and both parasite lines ΔHspJ62-I and ΔHspJ62-II were unable to form gametocytes. Microscopic images displayed a complete absence of gametocytes in the ΔHspJ62 parasites compared with the wild type (Fig. 4C). These results indicate that the HspJ62 protein is essential for gametocyte formation and is the likely reason for rapid asexual growth of knockout strains, as parasites that otherwise would have committed for sexual growth are now diverted towards asexual growth. Increased growth of mutant parasites when they are unable to form gametocytes has been observed earlier^34,35^. Our observations also showed increased growth in ΔHspJ62 parasites as their sexual stages were affected.

The sensitivity of gametocytes to chloroquine during their development and maturation has previously been studied. Although asexual erythrocytic parasites and immature gametocytes of *Plasmodium* are killed by chloroquine, mature gametocytes are not^36^. To confirm the defect in gametocyte formation due to HspJ62 gene deletion, groups of mice were infected with ΔHspJ62 and wild-type parasites. The parasites were allowed to grow for three days for gametocyte maturation. The mice were then treated with chloroquine (10 mg/kg/day) orally for three days. Posttreatment, Giemsa-stained blood smears were used for analysis. We observed that asexual stages were cleared in both the WT and knockout strains. Nevertheless, the WT mice showed the presence of mature gametocytes after 3 days of treatment, while ΔHspJ62 parasite-infected mice showed no gametocytes, confirming the defect in gametocyte formation (Fig. 4D).

To doubly ensure an accurate analysis of the absence of mature gametocytes in ΔHspJ62, we investigated the egress and exflagellation of male gametocytes and ookinete conversion starting from the same numbers of asexual stage parasites. For this, the numbers of male exflagellation centers were counted, followed by monitoring cultures for ookinete formation. The results showed no exflagellation centers in the ΔHspJ62 lines (Fig. 4E). In vitro ookinete conversion was also severely affected, and the mature ookinete number displayed complete abrogation in the ΔHspJ62 line compared to the wild-type parasite (Fig. 4F). Furthermore, mosquito feeding assays were set up to determine the pervasiveness of infection by counting the number of oocysts. We observed that the mean number of oocysts per midgut was reduced from 160 ± 50 in those fed wild-type parasites to zero (complete absence) in those fed ΔHspJ62 parasites (Fig. 4G). None of the sporozoites were found in the salivary glands of mosquitoes fed on mice infected with ΔHspJ62 parasites. In conclusion, the absence of ookinetes and oocysts in mosquitoes fed ΔHspJ62 parasites is due to the nonformation of gametocytes and zygotes.

### HspJ62 knockout leads to the downregulation of sexual stage-specific genes

**Figure 5:**
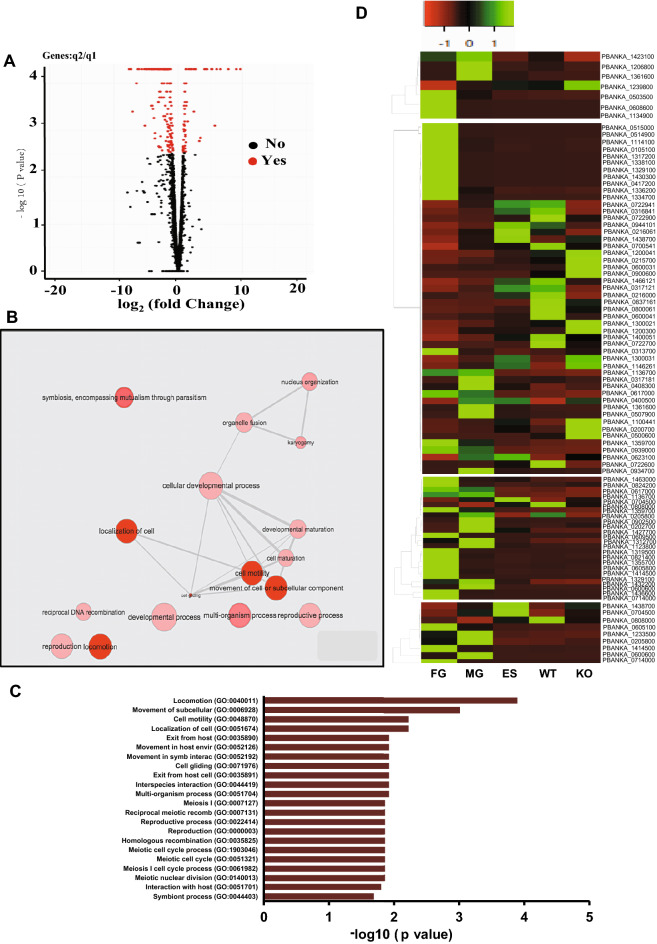
Transcriptomic Analysis of the ΔHspJ62 parasitic line. (**A**) Volcano plot of DEGs between the ΔHspJ62 and WT groups with log2 (fold change) as the x-axis and log10 (p value) as the y-axis. The volcano plot was made according to the gene expression level. The red dots indicate significant differentially expressed genes, and black dots indicate nonsignificant differentially expressed genes. (**B**) The ''Interactive graph'' view of REVIGO; bubble color indicates the user-provided p-value, and bubble size indicates the frequency of the GO term in the underlying GOA database. Highly similar GO terms are linked by edges in the graph, where the line width indicates the degree of similarity. Node initial placement was determined by a '’force-directed'’ layout algorithm that aims to keep the more similar nodes closer together. (**C**) Pathway enrichment analysis-The significant pathway for differentially expressed genes between *Δ*HspJ62 KO and control groups. Benjamin value is represented in the logarithm. (**D**) Heat Map. *FG* female gamete, *MG* Male gamete, *ES* erythrocytic stages, *WT* wild type, *KO* HspJ62 knockout.

**Figure 6:**
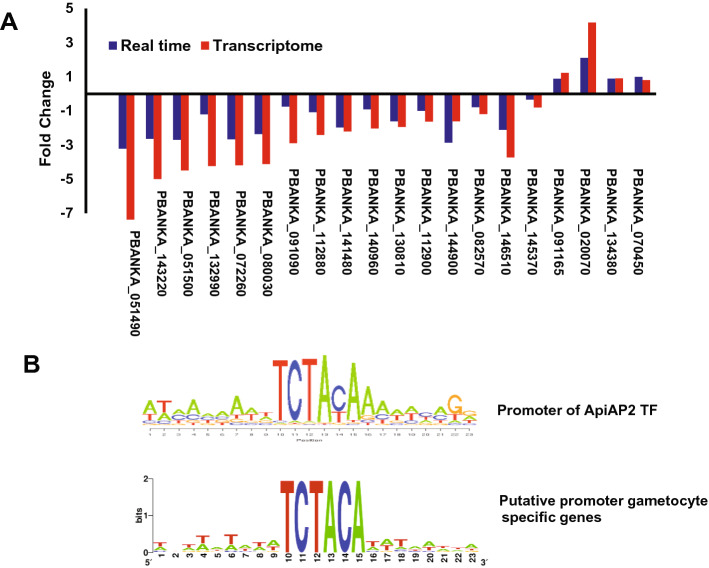
Transcriptomic Analysis of the ΔHspJ62 parasite line. (**A**) For validation of NGS data, qPCR was performed on 20 randomly selected up/downregulated genes from independent biological replicates of the experiment. GAPDH was taken as housekeeping genes. Blue and brown bars represent the fold changes (log10 scale) in P. berghei genes observed by qPCR and NGS, respectively. (**B**) The transcriptional regulatory motif of eight bases, i.e. **TCTANAAA** was identified upstream of the first ATG codon in gametocyte-specific genes.

To better understand the role of the HspJ62 protein in gametogenesis, we performed high-throughput RNA sequencing of the erythrocytic stage of ΔHspJ62 and compared it with that of the WT parasite. We obtained approximately 50 million paired-end reads, with a total length of more than 4 gigabases (Gb) per sample. Approximately 90% of the reads were mapped to the *P. berghei* reference genome (PlasmoDB Release 33), as shown in Table 1. We identified the differentially expressed genes by comparing the transcription between the WT and ΔHspJ62 lines. The transcripts in duplicate samples showing more than or equal to a 1.5-fold change and a P-value < 0.05 were selected. We found that out of 275 differentially expressed genes (Supplementary Table 2), 214 genes, including ApiAP2 (PBANKA_1453700), were significantly downregulated, while 61 genes were significantly upregulated (Fig. 5A). To achieve a functional annotation of the differentially expressed genes, we performed Gene Ontology (GO) in terms of biological processes, while the enrichment analysis of 275 DEGs was visualized by REVIGO. GO enrichment analysis found that most of the differentially expressed genes were primarily involved in locomotion (GO:0040011), movement of subcellular (GO:0006928), cell motility (GO:0048870), reproductive processes (GO:0022414), reproduction (GO:0000003), etc. that have significant P-value (< 0.005) (Fig. 5B and C). Furthermore, we compared the blood stage WT and ΔHspJ62 transcriptome data with the already known stage-specific transcriptome data^37^. We found that most of the downregulated genes in transcriptomic analysis were specific to either male or female gametocytes. The expression level of these genes is shown in a heat map (Fig. 5D). Apart from downregulated genes, we found that approximately 22% of differentially expressed genes were upregulated by more than 1.5-fold and were specific to asexual erythrocytic growth of parasites. To validate our transcriptome data, we performed qPCR with 20 randomly selected (both up/downregulated) genes using independent biological replicates of the experiment. The qPCR results correspond with the NGS data (Fig. 6A, for primer sequences please refer to Supplementary Table 1).

**Table 1:** Detail of mapped reads with the *P. berghei* genome.

Sample code	Sample name	Left reads		Right reads		% Total read mapping rate	% pair alignment Rate
		Input	Mapped	Input	Mapped		
7977	*Pb*HspJ62-KO	19,355,458	17,933,029	19,355,458	17,800,315	92.3	89.1
7978	*Pb*HspJ62-KO	25,528,700	24,040,739	25,528,700	23,817,699	93.7	90.7
7979	WT	24,204,342	22,651,763	24,204,342	22,460,127	92.8	89.9
7980	WT	25,851,476	24,058,393	25,851,476	23,830,640	92.6	89.2

To identify possible interacting partners of HspJ62 protein, we performed a pull down assay from whole parasite lysate using recombinant GST-HspJ62 protein immobilized on beads. After elution, the bound proteins were identified by LC MS/MS analysis; the most likely interacting partners are summarized in Supplementary Fig. 4. GST protein-bound beads were used as a negative control to eliminate nonspecific proteins or proteins with affinity for the GST tag (Supplementary Fig. 4A). We found that interacting proteins belonged predominantly to three major types of protein families, i.e. Transcription factors, RNA binding proteins and histone proteins interacted with the transcription factor ApiAP2 (PBANKA_1453700) (Supplementary Fig. 4B). To verify the interaction between ApiAP2 and HspJ62, we performed a far western blot with ApiAP2 recombinant protein using HspJ62 protein as a probe. ApiAP2 protein was used as prey, while purified recombinant HspJ62 protein was used as bait. ApiAP2 protein was run on SDS-PAGE gels and transferred to nitrocellulose membranes. The membrane was incubated with purified bait (recombinant HspJ62) protein overnight followed by the addition of anti-HspJ62 antibody. The secondary antibody (HRP-labeled anti-rat IgG) was used prior to developing the membrane by DAB using a standard western blot protocol. BSA protein was used as a negative control, and HspJ62 was used as a positive control (Supplementary Fig. 4C,D). The bimolecular interactions were also confirmed by ELISA. For this, the recombinant ApiAp2 protein was coated on a 96-well plate and incubated with recombinant HspJ62 protein. Anti-HspJ62 antibodies (polyclonal) were used to confirm the interaction. BSA was used as a negative control (Supplementary Fig. 4E).

ApiAP2 is a family of Apicomplexa-specific proteins containing AP2 binding domains found in malaria parasites. The proteins of this family include sequence-specific transcription factors that are key regulators of development. However, the function of the majority of ApiAP2 genes is unknown. The molecule of interest, ApiAp2 (PBANKA_1453700), possesses an AP2 domain for interaction with DNA and likely regulates the expression of many genes. However, this gene needs further functional characterization. Studies on the DNA binding of the ApiAP2 family of transcription factors show that the AP2 domain of ApiAP2 has binding specificity for unique DNA sequences. These sequence motifs are found in the upstream regions of a distinct set of genes, and these correspond to the sequence of eight bases ‘TCTANAAA’^38^. We screened the promoter region within 1000 bp upstream sequences from the first ATG codon in downregulated genes obtained from transcriptomic data (Table 2). Promoter analysis showed that 49 gametocyte-specific genes have these eight conserved base sequences TCTACATA in their promoter regions (Fig. 6B). We hypothesize that the ApiAP2 transcription factor binds to this conserved sequence and regulates the expression of these 49 gametocyte-specific genes.

**Table 2:**
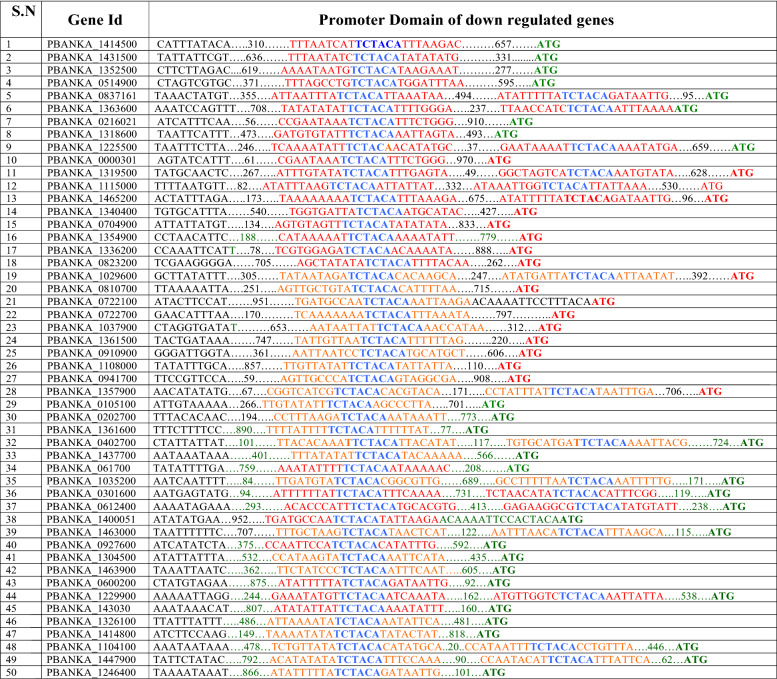
List of gametocyte specific down regulated genes whose promoter region contains signature sequence "TCTACA".

## Discussion

HspJ62 is a putative heat shock protein containing a DNAJ domain. Our results show that protein expression increases markedly at elevated temperatures or during heat shock. In fact, a remarkable increase in the expression intensity of HspJ62 was observed even when compared with HSP70 protein. HSP70 is known to be induced during heat shock and was used as a positive control in our experiments. Consistent with previous knowledge suggesting that HSPs are induced at high temperatures, the HspJ62 protein is classified as an HSP protein.

The exported proteins are involved in host modulation for parasite benefit. In silico analysis of HspJ62 showed the presence of two PEXEL motifs in the N-terminus of this protein. Our immunofluorescence results point to the possible export of HspJ62 into the RBC cytoplasm. However, the export of this molecule across the parasitophorous vacuole membrane (PVM) in the host cytoplasm needs to be confirmed by using PEXEL mutants. Transcript analysis and immunofluorescence assays showed that the protein was expressed during all stages of the parasite life cycle, i.e., the exoerythrocytic, erythrocytic and mosquito stages. The constitutive expression of HspJ62 protein indicates that the protein could have an important role in all *Plasmodium* stages. However, the subcellular localization and the mechanism of export of HspJ62 in host cells have yet to be determined.

Studies have shown that gametogenesis is increased under different stress conditions, such as ER stress and heat stress, indicating that there could be possible functional links between stress-induced protein synthesis and gametogenesis. To understand the functional role of HspJ62, we generated an HspJ62 gene-disrupted line of *P*. *berghei*. We closely monitored the growth and proliferation of two independent clones of the knockout parasite line (ΔHspJ62). We observed that parasites lacking HspJ62 showed faster growth in the asexual erythrocyte stage, while the development of sexual stage growth was completely arrested. We hypothesize that due to aberrant gametogenesis in knockout parasites, these parasites were unable to form subsequent sexual stages, and all the merozoites formed were directed to form asexual parasites, leading to faster asexual growth in the knockout parasite line than in the wild type. Furthermore, when the mice were infected with WT and ΔHspJ62 parasites followed by chloroquine drug treatment that affects all asexual stages of parasite while having no effect on sexual stages, we observed a complete clearance of parasites in mice infected with ΔHspJ62 parasite but not in the wild type, thus providing evidence for the absence of gametocytes formation in ΔHspJ62 line. Moreover, none of the mosquito stages of parasites were formed upon feeding with KO blood stage parasites. These experiments revealed that the HspJ62 protein is indispensable for gametocyte formation and plays a profound role in the development of sexual stages of the *Plasmodium* life cycle.

The genes that control gametocyte development are being identified and characterized. The deletion of HspJ62 triggers multiple changes in the transcription and translation of parasite proteins, and its cumulative effect results in the absence of gametocytes. To understand the role of the HspJ62 protein in parasites, we conducted a pull-down assay and identified the protein interacting partners. Our results suggest the interaction of HspJ62 with the transcription factor ApiAP2. These results were further confirmed by far western blot analysis and direct ELISA. However, reverse Co-IP using anti-ApiAp2 antibodies (not done yet) will confirm the interaction. Previous studies have revealed that members of the *Plasmodium* Apetala2 (AP2) family of proteins are self-regulatory transcription factors that play critical roles in gametogenesis^36,39^. In fact, the *Pf*AP2G protein has been recognized as a master regulator controlling the transition of parasites to the sexual stage in *P. falciparum*^40^. This is based on the observations that AP2-G positively regulates the expression of a number of early gametocyte genes. Moreover, stage-specific RNA sequencing data show high expression of ApiAP2 (PBANKA_1453700) during the schizont stage of *Plasmodium* compared to other asexual blood stages. Schizonts represent a crucial stage of parasites, where the expression of some genes is important for the commitment to the sexual stage of *Plasmodium* parasites^35^. Our observations show that ΔHspJ62 exhibits a growth advantage over the wild-type parasites, likely due to the absence of sexually committed merozoites in the schizonts^36^.

To obtain a broader picture of the functional role of HspJ62, we performed transcriptomic analysis of knockout versus WT parasites using bidirectional RNA sequencing. Analysis of transcriptomic data showed that gene knockout had a remarkable effect on the parasite transcriptome, with 272 transcripts differentially expressed by more than 1.5-fold. The gametocyte-specific genes were significantly downregulated in blood stage cultures of knockout parasites. In-depth analysis of transcriptome data revealed that the expression of 214 genes (78% of DEGs) decreased by more than 1.5-fold in HspJ62 knockout parasites. These DEGs included gametocyte-specific genes such as male development gene 1 (MDG-1), NIMA-related kinase 3, P25, secreted ookinete protein, and LCCL domain-containing protein. The transcriptional repression of these genes led to an inability to form gametocytes, the merozoites formed asexual stages, and we observed an upregulation of genes specific for asexual stages. Approximately 61 asexual stage-specific genes (22% of DEGs) were upregulated 1.5-fold in the ΔHspJ62 parasite, which included reticulocyte binding protein, Nop52, tryptophan rich protein and PIR family proteins. Asexual gene-specific upregulation is potentially related to the rapid growth of these knockout parasites in mice. AP2 domain-containing ApiAP2 proteins are known to be present in all Apicomplexa. The AP2 domain binds to a large variety of DNA sequences, while the proteins of this family generate stage-specific gene expression patterns. A number of genes from this family have been studied functionally and have been shown to play key roles in the life cycle of parasites while being critical for gametogenesis, ookinete development, sporozoite formation and liver stage maturation. Our transcriptome analysis of HspJ62 knockout parasites detected changes in transcription that are primarily associated with the transmission phenotype. Our data also point to a particular role of the transcription factor ApiAP2 (PBANKA_1453700), which is 1.5-fold downregulated in the ΔHspJ62 parasite at the erythrocytic stage. The molecular mechanism that leads to a decrease in ApiAP2 transcription in the absence of HspJ62 has yet to be explored. ApiAP2 (PBANKA_1453700) may self-regulate its own expression, similar to Ap2-G family proteins^41^. ApiAP2 proteins are regulatory transcription factors that direct transcriptional modifications, leading to multiple transitions into morphologically distinct differentiation stages. The downregulation of ApiAP2 (PBANKA_1453700) disrupts transcriptional regulation of sexual stage genes and leads to disruption of gametocyte formation. ApiAP2 factors have regulatory roles and can both activate and repress the gene expression of downstream genes^42^. They interact with chromatin-binding proteins and target gene promoters by recruiting chromatin-modifying protein complexes^43^. This interaction represents one of the mechanisms through which ApiAP2 factors exert their regulatory functions^44^. Extending this concept, we wished to analyze the relationship among genes that share the response to altered expression of the ApiAP2 protein. For this, we conducted a promoter analysis of downregulated genes, where sequences were recognized as targets when peak summits were situated within the 1000 bp upstream of the initiation codon in DEGs. The analysis showed that 49 gametocyte-specific genes have a conserved promoter region containing nucleotide signatures (TCTAC/TA) that match the binding site (TCTACA) of the ApiAP2 transcription factor^38^. The downregulation of ApiAP2 makes it unavailable to bind the conserved promoter region on these 49 gametocyte-specific genes, leading to their downregulation. Due to the cumulative effect of the downregulation of gametocyte-specific genes, the parasite is unable to differentiate into sexual stages and continues to form asexual-stage parasites. In conclusion, gametocyte-specific genes downstream of ApiAP2 are downregulated significantly, leading to the inability of gametocyte formation in the ΔHspJ62 parasite line. Our findings thus highlight the critical role that HspJ62 plays in facilitating the transition from the asexual blood stage to gametocytes.

## Methods

### Experimental mice

Experiments related to animals were performed according to the protocol (NII/IAEC/534/19) approved by the CPCSEA controlled Institutional Animal Ethics Committee (IAEC), National Institute of Immunology (NII), India. The general care of the experimental animals for this study was in accordance with animal use guidelines for laboratory animals and in compliance with the Animal Welfare Act (Prevention of Cruelty to Animals Act 1960, Wildlife division, Ministry of Environment and Forest, India). The animals were obtained from NIIs in house breeding facility, India. Treatments were carried out in compliance with the ARRIVE guidelines. Eight-week-old C57BL/6 and BALB/c mice were used in experiments. The animals were sacrificed humanely by cervical dislocation under complete anesthesia using a mixture of ketamine and xylazine (ketamine 80 mg + xylazine 5 mg, per kg bodyweight) given intraperitoneally at the end of each experiment.

### Parasite cycle

Mice were used for feeding mosquitoes and for growing *Plasmodium berghei* ANKA and GFP-con 259cl2 (MRA-865) parasites, i.e. similar to WT. The parasite cycle was continued between mice and mosquitoes. For this, female mosquitoes were starved overnight followed by the next day feeding on mice infected with parasites. The mosquitoes were then kept at 19–20 °C with 70–80% relative humidity and a 12 h light cycle. Infected mosquitoes were fed 20% sucrose solution for 18–24 days post-infection blood meal. After 14 or 18 days, the mosquitoes were dissected to obtain oocysts from the midgut and sporozoites from the salivary glands.

### DNA construct preparation and generation of the PBANKA parasite line deficient in the PBANKA_0938300 gene (HspJ62)

The HspJ62 gene was replaced by a targeting construct cloned into a plasmid (pBC-GFP-hDHFR) using double homologous recombination. The targeting construct consisted of a reporter marker GFP and an hDHFR expression cassette (pyrimethamine resistance) placed between the 702 bp 5′UTR and 817 bp 3′UTR of the *Pb*HspJ62 gene (PBANKA_0938300) to form homologous gene sequences on either side of the cassette. The 5′UTR was cloned between the XhoI and SalI sites, while the 3′UTR was cloned between restriction sites NotI and SacI (primer details given in Supplementary Table 1). For this, the 5′UTR was amplified using genomic DNA of P. berghei. The digested amplicon was ligated between the XhoI and SalI sites of the plasmid. The plasmid was transformed into E. coli, and the positive clone was confirmed by restriction digestion and sequencing. After confirming the 5′UTR, the 3′UTR was also cloned similarly between NotI and SacI sites. The final construct was confirmed by double digestion and sequencing. Approximately 10 μg of plasmid was linearized using ScaI enzyme and electroporated into purified schizont stage of *P. berghei* ANKA strain using Lonza Nucleofector reagent (cat# VPA-1006) and U033 program in Nucleofector 2B device. The transfected parasites were immediately injected into mice via the intravenous route. After 24 h, mice were placed under pyrimethamine drug selection for three days. The mice were observed for 13–15 days, and the drug-resistant parasites were analyzed for GFP expression. The GFP-positive parasites were used for initial genotyping by diagnostic PCR. The GFP-expressing resistant parasites were single-cell cloned, and the deletion of the targeted region was verified by diagnostic PCR and Southern blot analysis.

### Southern blotting

ScaI-digested genomic DNA was run for 14 h on a 0.8% agarose gel at 30 V and then transferred overnight to a positively charged nylon membrane (Roche, Germany) by the capillary transfer method. The transferred DNA on the membrane was UV cross-linked by applying 120 mJ of energy to the nylon membrane. The probe was prepared using the amplicon of the 5’UTR of HspJ62 (702 bp) (primer details given in Supplementary Table 1) labeled with digoxigenin, following the instructions provided by the kit’s manufacturer (Roche, cat # 11585614910), and Southern blot was developed according to the protocol of the kit supplier.

### Ex-flagellation assay

The mice were injected with 10^6^ parasites intraperitoneally. Four days post infection, the parasites in blood were monitored for gametocyte formation. The mice were anesthetized, and parasite-infected blood was collected. Exflagellation of mature male gametocytes was induced by adding 20 μl of ookinete medium (RPMI 1640 supplemented with 25 mM HEPES, 50 μg/ml hypoxanthine, 2 g/liter NaHCO_3_, and 100 μM xanthurenic acid) to 10 μl of parasite-infected mouse blood. After ten minutes of incubation at 19 °C, exflagellation was observed in activated male gametocytes. The numbers of exflagellation centers were counted in both control WT-GFP and KO parasites using a fluorescence microscope (Nikon 80i, at 100X).

### In vitro ookinete conversion

Phenylhydrazine (1.2 mg, Sigma-Aldrich, USA) mixed in 1X PBS was injected into BALB/c mice intraperitoneally 3 days prior to challenge with *P. berghei* parasites. Approximately 2 × 10^6^ infected red blood cells (iRBCs) were injected into these mice intraperitoneally to initiate blood-stage infection. When the parasitemia reached approximately 3–5% approximately 3 days postinfection, the mice were anesthetized. The infected blood was diluted 10 times in ookinete culture medium (20% (v/v) FBS, 1 mg/l heparin, 50 mg/l streptomycin, and 50 mg/l penicillin in RPMI 1640 pH 8.5), cultured in a T25 flask filled with a gas mixture of nitrogen (90%), CO_2_ (5%) and oxygen (5%) and maintained at 19 °C. After 24 h, the slides were prepared, and ookinetes formed were counted.

## Counting of in-vivo oocyst and sporozoite load

BALB/c mice were injected with 2 × 10^6^ parasite-infected red blood cells (iRBCs) intraperitoneally to initiate the infection. The mice were anesthetized when the parasitemia reached approximately 5–8%, and mosquitoes were allowed to feed on them for 10 min. The mosquitoes were dissected on days 14 and 18 after feeding to measure the presence and number of oocysts and sporozoites, respectively. On day 14, approximately 40 midguts were individually visualized under a fluorescence microscope, and the numbers of oocysts were counted. On day 18, salivary glands were individually dissected from the remaining 40 mosquitoes and homogenized gently to release sporozoites, which were then counted in a hemocytometer to calculate the average number of sporozoites per mosquito.

### Parasite infection and parasitemia

BALB/c mice were intravenously injected with 1 × 10^4^ blood stage parasites. Parasite growth was monitored by daily parasite counting in approximately 5000 erythrocytes until mice died. For this, thin blood films made from mouse blood were stained using Giemsa staining. To screen the ability of parasites to undergo sexual development and sporogony, groups of BALB/c mice were infected with 2 × 10^6^ knockout or wild-type blood stage parasites intraperitoneally and monitored for gametocyte formation by analyzing Giemsa-stained thin blood films under a light microscope.

### Transcript profiling

Total RNA was isolated from different parasitic stages, and cDNA was prepared by performing reverse transcription from RNA using random hexamer primers following the protocol mentioned in the kit (Bio-Rad #1708891). qPCR was performed using a gene-specific primer pair (primer details given in Supplementary Table 1). To check target gene expression under heat stress, infected blood at approximately 3% parasitemia was collected from mice and cultured at 37 °C and 41 °C in vitro. The parasites were harvested at 3 h and 6 h after heat stress.

### Cloning, expression and purification of HspJ62 protein

The *E. coli* codon-optimized (Entelechon, Germany) HspJ62 gene (amino acids 2–424 of PBANKA**_**0938300) was cloned (primer details given in Supplementary Table 1) between the XhoI and BamHI restriction sites of the pGEX-6P1 vector (GE Healthcare). The vector contains a glutathione *S*-transferase (GST) tag that is located at the N-terminus of the fusion protein. The calculated fusion protein size is 72,530 Da. The HspJ62 gene cloned vector was transformed into *E. coli* strain BL21-CodonPlus-RIL (Stratagene), and the cells were grown in culture medium containing 100 μg/ml ampicillin at 37 °C. Isopropyl 1-thio-β-d-galactopyranoside (IPTG, BioSynth Inc., USA) was added to a final conc. of 1 mM, was used to induce the culture when its O. The D_600_ reached between 0.5 and 0.6 and was further incubated at 18 °C for 22 h. The cells were harvested by centrifuging the culture at 10,000 rpm for 10 min at 4 °C and suspended in cell lysis buffer containing 0.025 mg/ml lysozyme (Bio Basic Inc. Canada) and 1 × Protease Inhibitor mixture (Roche, Germany). The culture was sonicated at 4 °C (ice-cold H_2_O) for 10 min at an amplitude of 35 (Qsonica Sonicator, Microprobe). The lysed sample was centrifuged at 13,000 rpm for 20 mint at 4 °C in an R247 rotor (REMI-CPR30 centrifuge). GST beads (GE Healthcare) were added to the cleared lysate and kept for binding at 4 °C for 8 h. Following binding, beads were loaded on a column and washed with 0.2 mM reduced glutathione-containing buffer. Elution buffer containing 25 mM reduced glutathione was used to elute the protein.

### Rat immunization and antibody generation

Four-week-old SD rats (n = 2) were primed with recombinant HspJ62 protein (50 μg) emulsified in complete Freund’s adjuvant. The first boost was given 14 days post priming with protein (30 μg/rat) emulsified in incomplete Freund’s adjuvant. The two subsequent boosts were followed using the same amount of protein as in the first boost at one-week intervals each. Preimmune sera were collected two days before immunization from the same rat. Blood was collected from the retro-orbital sinus one week after the second boost and from cardiac puncture one week after the third boost. The blood was allowed to clot at room temperature for 1 h and centrifuged at full speed (Eppendorf model 5415R), and serum containing antibodies was collected and stored at − 20 °C.

### ELISA

The recombinant HspJ62 protein was coated in each well of a 96-well plate, incubated at 37 °C for 3 h and then stored at 4 °C. The next day, the wells were washed three times with 1X PBST and three times with 1 × PBS and then blocked with 3% bovine serum albumin (BSA) for 1 h at room temperature. Serum collected from rats was added to the plate at increasing dilutions (100 µl/well) and incubated at 37 °C for 2 h. The plate was washed thrice with 1X PBST and 1X PBS. After washing, 100 µl HRP-conjugated goat anti-rat IgG antibodies (Invitrogen) diluted 1:4000 was added to the plate and further incubated at 37 °C for 1 h. The plate was washed again with PBST, followed by the addition of 50 µl of TMB substrate (Sigma). The color was allowed to develop for 10 min. The reaction was stopped by adding 50 µl of 2 mM H_2_SO_4,_ to each well. The plate was read immediately in a plate reader at 490 nm.

### Immunofluorescence assay (IFA)

The parasites were collected at different stages of their life cycle. Erythrocytic stage parasites were collected from mouse blood; ookinetes were collected from in vitro culture; oocysts and sporozoites were collected from the mosquito gut and salivary gland, respectively; and liver stage parasites were collected from in vitro infected HepG2 cells. Slides of each stage were prepared, fixed with 4% paraformaldehyde for 15 min and permeabilized with 0.1% saponin (Sigma) for 10 min. Samples were blocked using 3% BSA, followed by incubation in anti-HspJ62 antibody (1:5,00 dilution) at room temperature for 2 h. The slides with samples were washed and incubated with Alexa Fluor-conjugated goat anti-rat IgG (1:1000 dilution) for 1 h. After three washes, parasite nuclei were stained with 4′,6′-diamidino-2-phenylindole (DAPI, Invitrogen, USA). Slides were mounted with ProLong Gold antifade reagent (Invitrogen USA), and images were captured with a fluorescence microscope (Zeiss Axio-Imager M2).

### Pull-down assay

The infected RBCs were collected from mice, and RBCs were lysed using 0.2% saponin. The free parasites obtained were washed thrice with 1X PBS and lysed in IP lysis buffer (150 mM NaCl, 250 mM Tris, 1 mM EDTA, 5% glycerol, 1% NP-40, pH 7.4), followed by centrifugation at 12,000×*g* to obtain a clear lysate. The lysate protein content was quantified using a Pierce BCA protein assay kit. The beads coated with recombinant HspJ62 protein were added to parasite lysate and incubated at 4 °C under mild agitation for 12 h (10 μg of recombinant HspJ62 protein on beads was incubated with 100 μg of total parasite proteins). The beads were washed three times in IP buffer, and the bound proteins were eluted from the beads using elution buffer (25 mM reduced glutathione, 150 mm NaCl, 100 mm Tris, 0.5 mm EDTA, 10% glycerol, pH 8.5). Eluted proteins were treated with the enzyme trypsin in solution. An AB SCIEX MALDI-TOF/TOF-4800 mass spectrometer coupled to nano-LC 1000 was employed to analyze peptides generated upon trypsin treatment. The raw data obtained during nano LC–MS/MS analysis were processed and searched with *the P. berghei* PlasmoDB database.

### In-solution trypsin digestion

The proteins in the samples were treated before digestion with trypsin in solution. The samples were vacuum dried and volume compacted to nearly 100 µL in 50 mM ammonium bicarbonate (Sigma) buffer at pH 7.8, reduced using 10 mM DTT (final concentration) and alkylated with 40 mM iodoacetamide (Sigma Aldrich, USA) for 1 h in the dark at RT. The samples were then treated with sequencing grade trypsin (Promega) at a ratio of 1:50 (w/w) trypsin:protein and placed in a water bath at 37 °C for 18 h for complete digestion. Subsequent to digestion, the peptides were acidified in 0.1% formic acid.

### Far Western blot analysis

The predicted functional domain of the ApiAP2 protein (amino acids 708–1485 of PBANKA_1453700) was cloned into the pET28a vector, purified recombinant ApiAP2 protein was used as prey, and purified HspJ62 protein was used as bait using a modified protocol published earlier^45^. ApiAP2 protein was run on SDS-PAGE gels and transferred to nitrocellulose membranes. The denatured protein was renatured on the membrane in buffer (10% glycerol, 100 mM NaCl, 100 mM Tris pH 7.5, 0.001 EDTA, 1% Tween-2-, 6 M guanidine–HCl, 2% milk powder, 0.001 DTT) by gradually reducing the guanidine–HCl concentration from 6 to 0.1 M while rocking at room temperature. The membrane was washed three times with AC buffer [100 mM NaCl, 20 mM Tris (pH 7.6), 0.5 mM EDTA, 10% glycerol, 0.1% Tween-20, 2% skim milk powder and 1 mM DTT]. After blocking with 5% skimmed milk, the membrane was incubated with purified bait (recombinant HspJ62) protein overnight at 4 °C. The membrane was washed, added to buffer containing anti-HspJ62 antibody and incubated at RT for 2 h. The membrane was washed once again and incubated with secondary antibody (HRP-labeled anti-rat IgG) for one hour at 37 °C. The antibody concentrations used were 1:1000 anti-HspJ62 antibody and 1:5000 secondary antibody (HRP-labeled anti-rat). The membrane was developed by DAB using a standard western blot protocol.

### RNA isolation, library preparation, sequencing

Infected blood was collected from mice and passed though Plasmodipure (Euro Proxima, Netherlands, cat# 8011) to remove WBCs. The purified RBCs were lysed in 0.2% saponin, and the free parasite pellet was washed three times with PBS to obtain a pellet devoid of any RBC contamination. The parasite RNA was isolated using Trizol. RNA extraction and Illumina mRNA sequencing were performed in duplicate. For this, total RNA was isolated and quantified using a NanoQuant-M200 spectrophotometer (Tecan, UK). The RNA integrity number (RIN) was employed to check the quality of RNA, and it was more than 7. The mRNA was purified, subjected to cDNA synthesis and fragmented, and for adapter ligation, a single ‘A’ base was added at the end. The purified products were enhanced with PCR to make the final cDNA library. The quality of the cDNA library was analyzed on a Bioanalyzer, and deep sequencing of validated cDNA libraries was achieved by the Illumina HiSeq 1000 platform using manufacturer guidelines.

### Data processing

The Fast QC quality control tool (http://www.bioinformatics.babraham.ac.uk/projects/fastqc/) was used to check the quality of raw reads, which were then cleaned by using TrimGalore software (http://www.bioinformatics.babraham.ac.uk/projects/trim_galore). Subsequently, TopHat2 software was used to map transcriptome data with the reference genome (PlasmoDB Release 33). Nonexpressed contigs (with FPKM = 0) were excluded from this transcriptomic analysis. Raw read count data were used to identify differentially expressed genes using edgeR^46^.

### Differential expression

Gene expression differences in all the different experimental groups were analyzed employing Cuffdiff (version 2.2.1). Specific groups were matched with linear contrasts, and the P-values were optimized by multiple testing (i.e., false discovery rate). A P value (FDR) below 0.005 and a minimal log FC of 1.5 were considered differentially expressed^47^.

### Gene ontology enrichment

Differentially expressed gene (DEG) functional enrichment of gene ontology (GO) analyses was performed using the REVIGO tool in conjunction with the GO annotation available from PlasmoDB^48^ The analysis was based on a gene set of interest compared to all annotated genes using the “weight” algorithm with Fisher’s exact test (both implemented in REVIGO). A P-value of below 0.05 was considered significant.

### Validation of NGS data by real time PCR

NGS data was validated by RT-PCR. A random set of 20 differentially expressed genes was selected. 18S rRNA and GAPDH were used as housekeeping gene controls. The primers (primer details given in Supplementary Table 1) against each of these genes were designed using Primer3 software and synthesized. cDNA was made from independent biological replicates of WT and HspJ62-KO parasite lines of blood stage RNA. All reactions were performed on an Eppendorf RealPlex detection system using SYBR Green PCR master mix. The fold change in each selected gene was determined using the 2 ^−ΔΔ^Ct method.

### Contribution to the field statement

This work has provided insights into the critical role of a heat shock protein and the processes underlying gametocyte commitment and development. HspJ62, a putative chaperone molecule, is essential for the development of *P. berghei* sexual stages. The transgenic mutant *P. berghei* parasite line (ΔHspJ62) was unable to make gametocytes. This study characterizes HspJ62 protein as a fertility factor, as parasites lacking it are unable to transmit to mosquitoes. Our study adds an important contribution to ongoing research aimed at understanding gametocyte differentiation and formation in parasites.

## Supplementary Information

Supplementary Information 1.

Supplementary Table 1.

Supplementary Table 2.

## Data Availability

All data is shown within the manuscript and figures. The raw data and the analysis details that support the findings of this study are available from the corresponding author upon reasonable request. All of the RNA-seq raw file data were deposited in NCBI under GEO Accession Number GSE168974.

## Abbreviations

*P. berghei*: *Plasmodium berghei*
HspJ62: Heat Shock family J domain containing 62 kDa protein
