# Supplementary material for: Essential role of a Plasmodium berghei heat shock protein (PBANKA_0938300) in gametocyte development
Source: Sci Rep. 2021 Dec 8;11:23640. doi: 10.1038/s41598-021-03059-4 (PMC8654831; doi:10.1038/s41598-021-03059-4)
Supplement: Supplementary file 1 — Supplementary Information 1. [file 41598_2021_3059_MOESM1_ESM.doc]

**Additional Information**

Supplementary Table 1: List of primers use in this paper.

Supplementary Table 2: List of DEGs

Supplementary figure 1: Protein purification and antibody generation

Supplementary figure 2: Mass spectrometry data of HspJ62 protein and control IFA.

Supplementary figure 3: PEXEL motif analysis and asexual blood stage IFA.

Supplementary figure 4: Interaction partners of HspJ62.

Supplementary figure 5: Raw images/full blot images

Supplementary figure 6: Raw images

**Supplementary Figure 1: Expression and antibody generation of recombinant HspJ62: (A)** Coomasie blue stained SDS-PAGE of HspJ62 protein after single step purification by GST-affinity chromatography. The gene was cloned in pGEX-6P1 vector, transformed in E.coli (BL21) and expressed as GST fused recombinant HspJ62 protein. M- Protein Mass ladder, Elution- GST column purified fraction of HspJ62.

(B) Representation of immunization schedule using recombinant HspJ62 protein rats.

(C) The estimation of antibody end point titer employing Enzyme linked immunosorbent assay (ELISA)

(D) Immuno blotting using monoclonal anti-GST antibody against the GST tagged purified HspJ62 protein fractions E1 and E2.

E) Immuno blotting using anti-HspJ62 antibody against the *P. berghei* parasite protein lysate.

C- uninfected blood lysate, PL- parasite infected blood lysate.

**Legends Supplementary Figure 2:**

(A) List of unique peptide sequence of purified recombinant HspJ62 protein as identified by mass spectrometry

(B) Pre-immune rat sera used as a negative control for immunofluorescence assay

**Legends Supplementary Figure 3:**

(A) Putative PEXEL motif as observed by manually analyzing the protein sequence

(B) Immunofluorescence assays to localize HspJ62, in the different asexual stages of parasites using anti-HspJ62 antibodies.

**
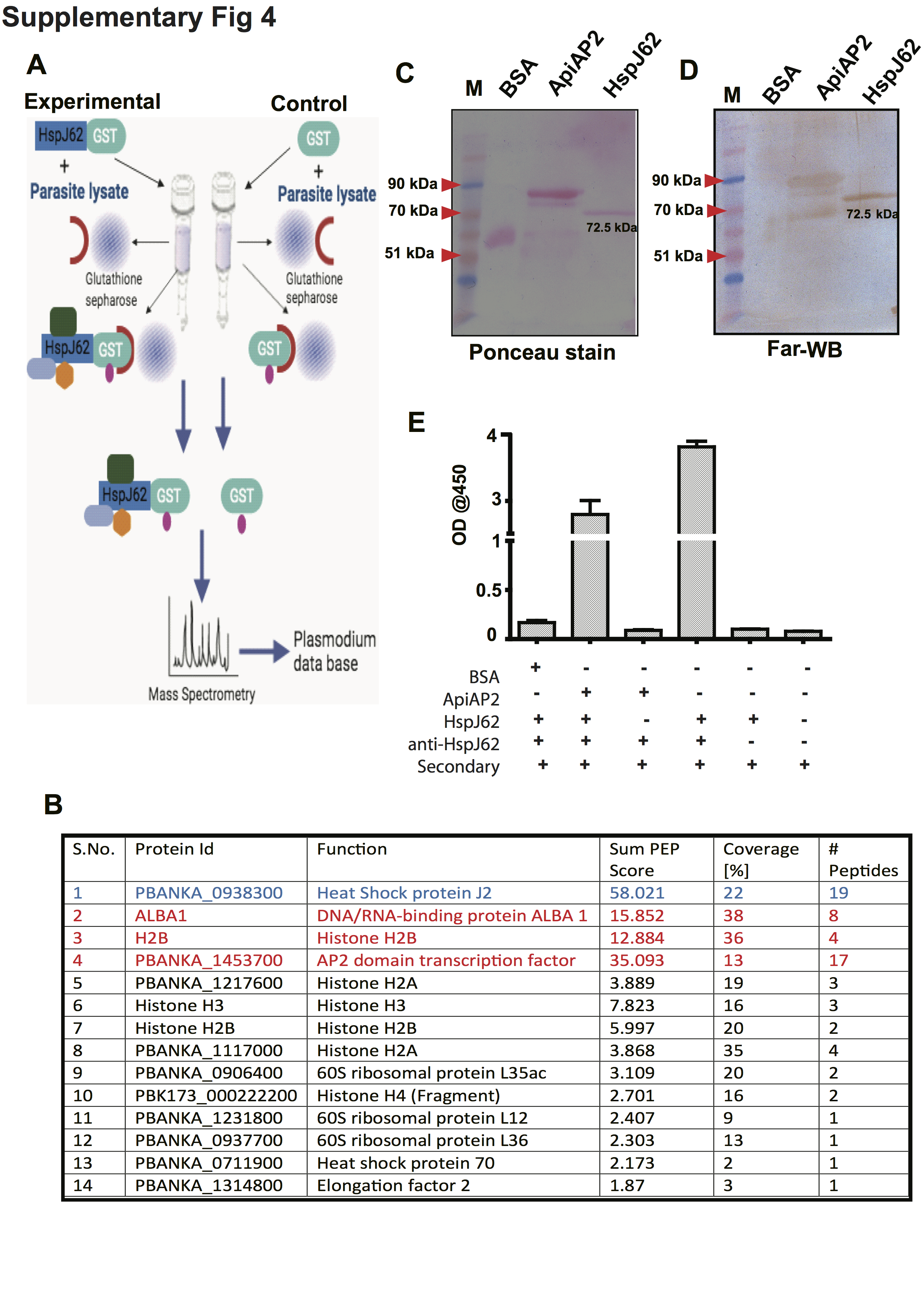
**

**Legends Supplementary Figure 4: Interaction of HspJ62 and ApiAP2 transcription factor:**

(A) GST-pull down assay; the Hspj62 protein was expressed as GST fusion protein in E.coli (BL21) and was incubated with parasite lysate. The associated proteins were recovered with glutathione-Sepharose resin

(B) Pulled proteins were subjected to Mass spectrometric analysis. The most probable interacting proteins (shown in red) identified by LC MS/MS analysis are summarized The proteins were scored for the percentage of coverage in which they were detected. Majorly 13 proteins, representing five protein classes (heat shock/chaperone, histones, AP2 domain transcription factor, DNA/RNA binding proteins and ribosomal proteins), were detected most frequently and potentially able to bind HspJ62.

(C) Recombinant HspJ62 and ApiAp2 protein were run on SDS-PAGE. M- Protein mass ladder, Lane 1 BSA (Bovine serum Albumin, negative control) Lane 2: ApiAP2 recombinant protein and Lane 3 HspJ62 recombinant protein. Separated proteins were transferred to nitrocellulose membrane. The blot was stained with Ponceau S stain and shown is the image of stained blot.

(D) The HspJ62 interaction with APiAP2. Protein was subjected to Far western blot analysis using anti-HspJ62 polyclonal antibody raised in rat. A membrane (as shown in Ponceau stained image, Supplementary. 4C)) was incubated with recombinant HspJ62 protein before allowing it to interact with anti-HspJ62 antibody. The blot was developed using DAB. Lane identity is same as in 4C.

(E) ELISA was performed to check the HspJ62 interaction with APiAP2 protein. BSA-Bovine serum Albumin, ApiAP2-ApiAP2 recombinant protein, HspJ62- HspJ62 recombinant protein, anti-HspJ62- polyclonal antibody against HspJ62, Secondary- anti mouse IgG coupled with HRP

M

ΔHspJ62-I

ΔHspJ62-II

WT

G

G-i
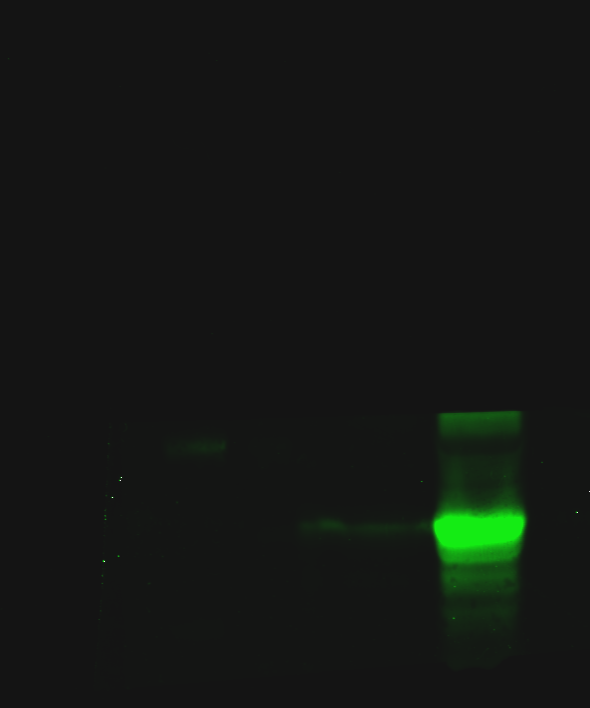


Probed with Anti- Parasite HspJ62

Western blot with antibodies labeled with IR-dye

M

ΔHspJ62-I

ΔHspJ62-II

WT

Probed with Anti- Parasite Aldolase

Gii
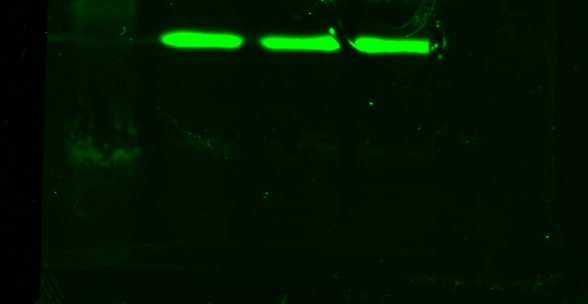


Western blot with antibodies labeled with IR-dye

H

M

ΔHspJ62-I

ΔHspJ62-II

WT


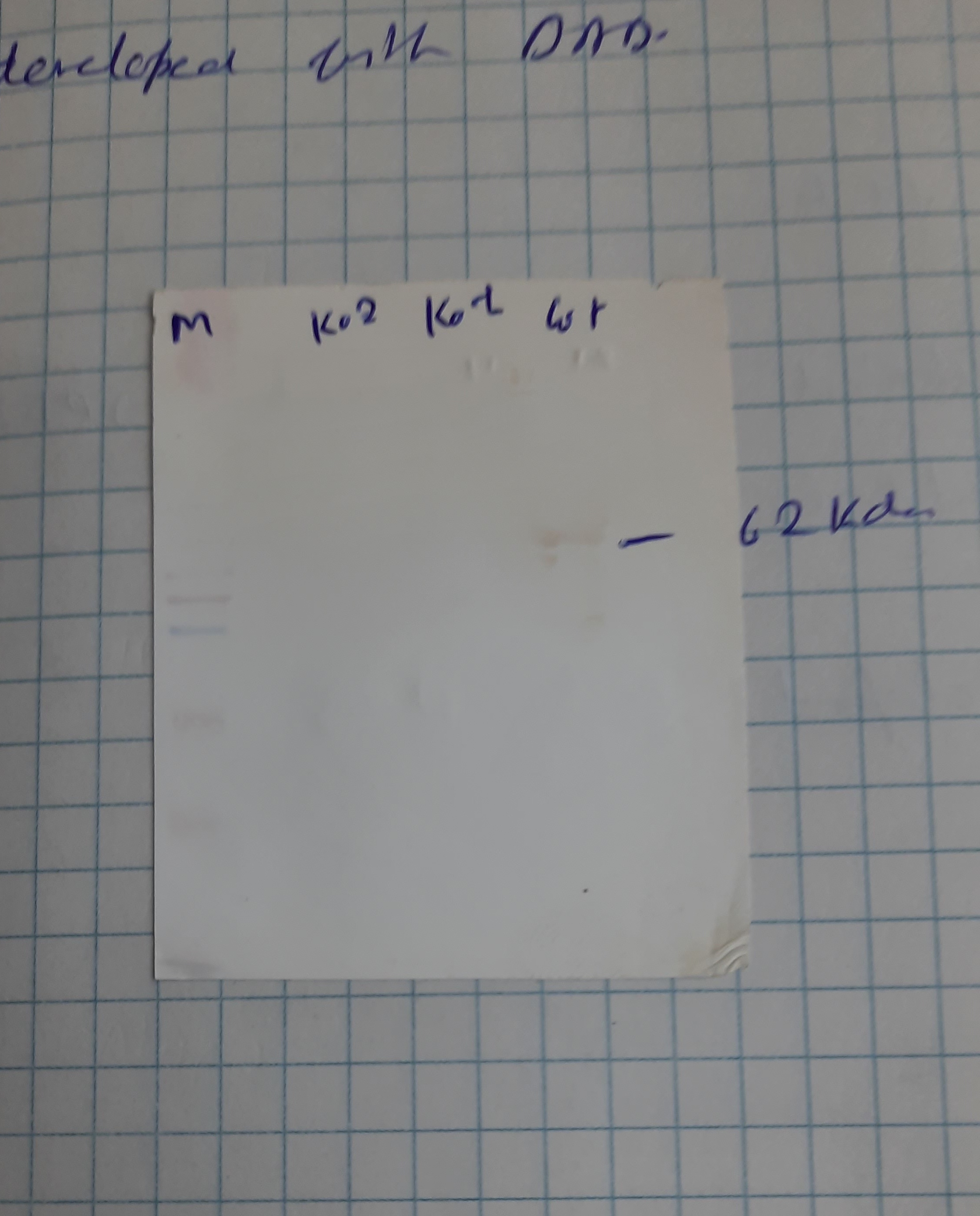


Probed with Anti-HspJ62

and developed with DAB

**Legends Supplementary Figure 5:**

(A &B) Full blot image of Figure 1B and its GAPDH control

(C) Full image of Ponceau stained membrane as shown in Supplementary 4C.

(D) Full western blot image of DAB developed image as shown in supplementary figure 4D.

(E) Full image of southern blotted membrane, along with the targeting construct as control, of figure 3D. WT- wild type gDNA, ΔHspJ62-I and ΔHspJ62-II- are the two knockout clones of HspJ62 knockout parasite lines.

(F) Full western blot image of wild type and HspJ62 knockout parasite lines lysate probed with anti-HspJ62 polyclonal antibody. Membrane was cut in two parts (top and bottom) and probed with the indicated antibodies (F). WT- wild type parasite lysate, ΔHspJ62-I and ΔHspJ62-II- are the parasite lysates from the two knockout clones of HspJ62 knockout parasite lines.

(G) Original scan (Infrared scanner, Licor) of the images shown in shown in Fig 3E and supplementary fig 5F. Gi -probed with anti-HSPJ62, Gii- probed with anti-parasite aldolase Membrane was cut in two parts before western with individual antibodies. Membrane size before cut was approximately 6 x5cm. WT- wild type parasite lysate, ΔHspJ62-I and ΔHspJ62-II- are the parasite lysates from the two knockout clones of HspJ62 knockout parasite lines.

(H) Membrane (a duplicate) identical to one shown in Supp 5-G was processed simultaneously with only anti-HspJ62 antibody and membrane was developed with chromogenic substrate DAB. Arrow indicates presence of protein in wild type but not in the HspJ62-knock outs. Full membrane is presented for comparison with the identical membrane that was cut in two parts for the scans shown in Supp 5-G. WT- wild type parasite lysate,KO-I and KO-II- are the parasite lysates from the two knockout clones of HspJ62 knockout parasite lines.

**Supplementary Figure 6**

**A**

**Replicate of loading control for Fig-3B**


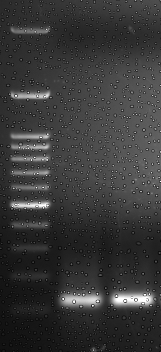


M WT KO

WT- wild type parasite, KO- HspJ62 knockout parasite line.

**
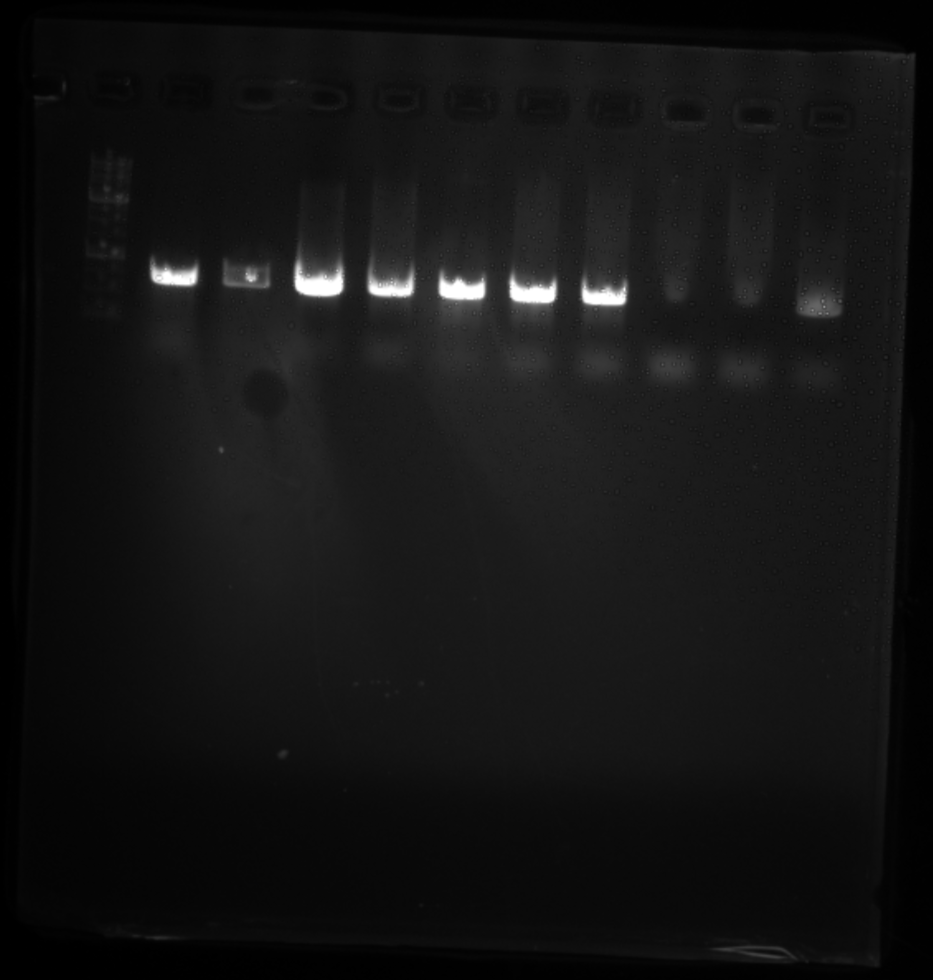
**

**B**

**Legends Supplementary Figure 6:**

[A] The full-image of loading control as shown in Fig-3B. This was just to check the quality of parasite’s genomic DNA.

[B] Full Image of loading control of Fig 3C
