# Supplementary material for: Essential role of a Plasmodium berghei heat shock protein (PBANKA_0938300) in gametocyte development
Source: Sci Rep. 2021 Dec 8;11:23640. doi: 10.1038/s41598-021-03059-4 (PMC8654831; doi:10.1038/s41598-021-03059-4)
Supplement: Supplementary file 2 — Supplementary Table 1. [file 41598_2021_3059_MOESM2_ESM.docx]

**Supplementary Table 1:** List of primers used in this paper

| **S.No.** | **Oligo Name** | **Oligo sequence (5’-3’)** |
| --- | --- | --- |
| 1 | 5’UTR FR | TGTCTCGAGGCAAGGCAAAATGACAATGTTC |
| 2 | 5’UTR RV | TGTGTCGACGACATGCGCTGAGGGAGTG |
| 3 | 3’UTR FR | TGTGCGGCCGCGCCATATTGTTGCACAACATAG |
| 4 | 3’UTR RV | TGTCCGCGGGTGGGTTAAAATCGGAAGTGTC |
| 5 | DP1 | CTTTACACACATAAAATGGCTAGTATG |
| 6 | DP2 | TCACGGCTGTGTGTATCAT |
| 7 | SP1 | GGCTAGGATTAAAACGAAATGCA |
| 8 | SP2 | CTTTAGGTATATCCGCAC |
| 9 | PbHSPJ62P F | TGTGGATCCGACTACAAAGATGACGATGACAAAAAC |
| 10 | PbHSPJ62P R | CCCTCGAGTCAATATTTTTTTTTGGTCAGTTCC |
| 11 | PbHSP70 (PBANKA_081890) FP | GCTGGTGCAATTGCTGGTTTA |
| 12 | PbHSP70 (PBANKA_0818900) RP | CCTGCTGTTGCATATACTTCA |
| 13 | 18sRNA FP | AAGCATTAAATAAAGCGAATACATCCTTAC |
| 14 | 18sRNA RP | GGAGATTGGTTTTGACGTTTATGTG |
| 15 | ApiAp2 (PBANKA_145370) FP | ACGAGGATCCATGGCACATGAACAAGGA |
| 16 | ApiAp2 (PBANKA_145370) RP | TGCACTCGAGAATGGATCTTCGGCAA |
| 17 | PbGAPDH FP | AATTAAAGAAGCATCTGAGGGTCCAC |
| 18 | PbGAPDH RP | TTGAATATCCCCATTCATTGTCATACC |
| 19 | PB051490F | ACCAGGGAAATTTTGGCTTA |
| 20 | PB051490R | CTCCTGTACCTGTGCATTTA |
| 21 | PB143220F | CAACATCAACCATAGGGTGTCTA |
| 22 | PB143220R | TAGCCATTGTCACTCCATTT |
| 23 | PB051500F | CGTGAATGATCCCCCAATAT |
| 24 | PB051500R | CCATCTTCACAGTTACACCTAT |
| 25 | PB132990F | GTTGCATCAAATGAATTAGCA |
| 26 | PB132990R | GCCCATTTGCATCAAAAGAT |
| 27 | PB072260F | AGAAGAAGCGGACAATCAAA |
| 28 | PB072260R | TTCTGTTCGAAGGTCATCAA |
| 29 | PB080030F | CCCGATTTAGTTGAAGGGAA |
| 30 | PB080030R | CGAATCATACGTACCGTCTT |
| 31 | PB091090F | CGCCCATATACACTTGGAAA |
| 32 | PB091090R | CGGGGTTAATACTGATACAT |
| 33 | PB112880F | AGCATACGAAGGACAGTGTA |
| 34 | PB112880R | CAGGTGCTATGCATAAGCTA |
| 35 | PB141480F | AAGCTTCAGCAGATTACGAA |
| 36 | PB141480R | CGACTTATCTCCGATAATGTCT |
| 37 | PB140960F | AGCGGATATGAAACTTGACA |
| 38 | PB140960R | TTGGTTCGTTCCTCTTTGAT |
| 39 | PB130810F | ACATAGCACCATTAGACATCA |
| 40 | PB130810R | CATTCTTTCAACTTCGCTCTT |
| 41 | PB112900F | CCGGTTGAAGATACACAAGA |
| 42 | PB112900R | TGAAGAGGTTCAAATGCTGA |
| 43 | PB144900F | TACAATTTCACGGCAATTCGAA |
| 44 | PB144900R | CCCCAGGATTGCTCATATTT |
| 45 | PB082570F | ATTCCCCGTCTATGTTAACT |
| 46 | PB082570R | CAGTCCAGTTTGATAATCCAT |
| 47 | PB146510F | CAACTACCCAGGTGGAAAAT |
| 48 | PB146510R | ATCCTCGTTATTGCCGATAT |
| 49 | PB145370F | AGGAGAAGCATGGGATAGAT |
| 50 | PB145370R | CCTACCCATCCCATTCTTTGT |
| 51 | PB091165F | GCCTCTTGGCTAAGATCAATTT |
| 52 | PB091165R | ATTAGTGCTAGGGTAAGGCA |
| 53 | PB020070F | CGATAGAGGGATATGCCTTGA |
| 54 | PB020070R | TCGAAGGTGTGCTATTTCTT |
| 55 | PB134380F | TTTGACGAAAACGAGATGGGAA |
| 56 | PB134380R | TCATTTGAACCTCGAAAGCT |
| 57 | PB070450F | ACAATCCTAATACCCCTCGA |
| 58 | PB070450R | TTTCGGAGTCATTTGCAGTA |
